# Experimental Determination of Detector-Relevant Properties of ZnSe(Al,O) for Beta Spectroscopy

**DOI:** 10.3390/s26175454

**Published:** 2026-08-28

**Authors:** Arjana Kolnikaj, Kelum A. A. Gamage, Olaoluwa Popoola, James Graham, Antonio Di Buono

**Affiliations:** 1 James Watt School of Engineering, University of Glasgow, Glasgow G12 8QQ, UK; 2 United Kingdom National Nuclear Laboratory, Sellafield, Seascale, Cumbria CA20 1PG, UK

**Keywords:** ZnSe(Al,O), scintillator characterisation, beta spectroscopy, light yield, refractive index, emission spectroscopy, decay time, SiPM, Sr-90, radiation detection

## Abstract

ZnSe-based scintillators are promising materials for compact beta spectroscopy, but the detector-relevant properties of ZnSe(Al,O) remain poorly documented and strongly dependent on dopant and defect structure. This work presents an experimental characterisation of a commercially supplied ZnSe(Al,O) crystal intended for an SiPM-based beta detector to Sr-90/Y-90 detection. A scanning electron microscope with energy dispersive x-ray spectroscopy (SEM-EDS) was used to verify the elemental composition and examine surface artefacts, while optical and scintillation properties were determined through density measurement, Brewster-angle refractive index measurement, UV-excited emission spectroscopy, Sr-90/Y-90 charge-spectrum analysis, and waveform decay fitting. The crystal was found to contain Zn, Se, Al, and O, with average atomic abundances of 50.3%, 44.0%, 1.2%, and 4.5%, respectively. The measured density was 5.31 g ${\mathrm{cm}}^{-3}$ and the refractive index was 2.574 at 650 nm. The emission spectrum peaked at approximately 581 nm, within the visible response range of silicon photodetectors. Sr-90/Y-90 beta measurements gave an estimated intrinsic light yield of approximately $7.69\times 10^{4}$ photons ${\mathrm{MeV}}^{-1}$, while exponential fitting of digitised waveforms gave a decay time of 9.41 μs. These results provide an experimentally determined parameter set for this ZnSe(Al,O) crystal and support its use in compact beta spectroscopy, while also highlighting the need for direct material characterisation before reliable detector simulation.

## 1. Introduction

Zinc Selenide (ZnSe) is a pure semiconductor material with a wide bandgap of 2.7 eV (at room temperature) and density of 5.3 g ${\mathrm{cm}}^{-3}$ [1]. ZnSe-based scintillators have emission wavelengths that match photodiodes well and have been used or proposed for detecting alpha, beta, gamma and, x-ray radiation. The optical and scintillation properties of ZnSe-based materials, such as emission spectrum, light yield, and decay kinetics, are strongly dependent on the choice of dopant. Un-doped ZnSe is widely used for the lenses of ${\mathrm{CO}}_{2}$ lasers due to its excellent photon transparency in the infrared (IR) range [2]. Other dopants such as tellurium (Te), cadmium (Cd), oxygen (O), aluminium (Al), and combinations of all four have been used for radiation detection. This study will examine ZnSe(Al,O). Currently, this crystal is part of a detector system to monitor Sr-90 in groundwater. The crystal is coupled to a silicon photo-multiplier (SiPM) and provides counts-based and spectral information. Part of the development of the detector has involved continuous refinement through both simulations and experimental validation. Accurate modelling of this system requires reliable material specifications. However, the crystal supplier provides no detailed information on dopant concentrations or optical properties. As a result, simulation parameters for the crystal were approximated using values from broader ZnSe literature. This motivates the present study, which first reviews reported properties of ZnSe-based scintillators, then describes the experimental methods used to measure the composition, density, refractive index, emission spectrum, light yield, and decay time of the supplied ZnSe(Al,O) crystal, before presenting these results as a detector-relevant parameter set for beta-spectroscopy modelling and prototype optimisation.

### Previous Work in Ionising Radiation Detection

The current available literature on ZnSe-based scintillators is limited and fragmented, particularly with respect to co-doped systems such as ZnSe(Al,O). A study comparing ZnS(Ag) and ZnSe(Al) for alpha particle detection reported improved energy resolution in ZnSe(Al) (∼30% compared to ∼70% for ZnS(Ag)) when measuring 5.5 MeV alpha particles from Am-241 [3]. This improvement was attributed to reduced self-absorption and more favourable optical transport in ZnSe-based materials. However, the study is limited to alpha particle interactions. Another study compared caesium iodide (CsI) with ZnSe(Te/O) for gamma ray detection using sources: Co-57, Am-241, Ba-133 and Cs-137 [4]. They reported that ZnSe(Te/O) is also suitable for x-ray detection and stated there was improved light collection efficiency with increasing crystal thickness. However, this improvement is inferred from higher signal amplitude rather than a direct measurement of light collection efficiency. The study quoted previously reported values for the light yield of ZnSe(Te/O) reaching a maximum of 80,000 photons/MeV; however, it experimentally measured 40,000 photons/MeV. Another property exhibiting significant variability was the decay time of the crystal. The study reports decay times of 3–15 μs for ZnSe(Al,O), alongside ∼2–8 μs for ZnSe(O) and ∼30–80 μs for ZnSe(Te). This wide spread indicates that decay time is not an intrinsic material constant and must be experimentally determined for a given crystal and detector configuration. This variability is further reflected in another study which directly measured the decay behaviour of ZnSe(O) [5]. The researchers grew ZnSe(O) single crystals and investigated how doping and annealing conditions influence their structural, optical, and scintillation properties. The decay response of the as-grown and annealed ZnSe(O) crystals exhibits two components, with time constants of 9 and 28 μs, and 4 and 12 μs, respectively.

A study investigated the photo-luminescence behaviour of ZnSe(Al,O) and its underlying recombination mechanisms [6]. The emission wavelengths were found to consist of two broad bands spanning approximately 575–650 nm, with decay behaviour described as non-exponential and governed by donor–acceptor pair recombination. The recombination rate depended strongly on defect and dopant concentrations, where higher aluminium content led to increased trap density and faster decay. These findings further demonstrate that the scintillation response of ZnSe(Al,O) is highly dependent on material composition and defect structure, reinforcing the need for direct characterisation.

While the literature discussed so far focuses primarily on material properties and gamma/alpha/x-ray detection, limited work has addressed the use of ZnSe-based scintillators for beta radiation detection. A study demonstrated the use of ZnSe(Te) for beta radiation detection through the development of a composite scintillator-photodiode detector [7]. The material showed peak emission between 610 and 640 nm, light yield up to ∼7.5 × $10^{4}$ photons/MeV, and decay times spanning ∼1–3 μs and ∼30–70 μs depending on the scintillation component. Beta sensitivity was reported as $5.5\;{\mathrm{cm}}^{2}$ using a ^90^Sr/^90^Y source, confirming the material’s suitability for beta detection. However, the study did not present a calibrated beta energy spectrum which limits its conclusions to detection capability rather than energy-resolved measurement. More recently, a study investigated the properties of ZnSe(Al) and ZnSe(Te) within the range of 77–295 K in terms of luminescence intensity and kinetics [8]. Emission was found to be peaked at 600 nm for ZnSe(Al) and 640 nm for ZnSe(Te), while decay kinetics were identified to be non-exponential with more than one type of recombination processes. Cooling down the crystals below 100 K significantly improved the scintillation properties, achieving twofold light yield increase.

This study will examine the ZnSe(Al,O) crystal, which is currently part of a detector system utilised to monitor Sr-90 in groundwater. Beta spectroscopy presents unique challenges compared to gamma or alpha detection; because beta particles are emitted with a continuous energy distribution rather than discrete energy peaks, accurate energy-resolved measurements require highly precise characterisation of the scintillator’s light yield, optical transport, and decay kinetics. The crystal in this system is coupled to a silicon photomultiplier (SiPM) to provide both counts-based and spectral information. Part of the development of this detector has involved continuous refinement through experimental validation and Geant4 Monte Carlo simulations. Accurate theoretical modelling of this system requires reliable material specifications. However, the crystal supplier provides no detailed information on dopant concentrations or optical properties, meaning simulation parameters were initially approximated using highly variable values from the broader ZnSe literature.

Overall, across the literature, key detector properties such as light yield, decay time, and emission characteristics show significant variation. Table 1 summarises the properties of interest to this study for each ZnSe-based scintillator material. The work presented here moves beyond reported values by presenting direct measurements of detector-relevant properties for a ZnSe(Al,O) crystal. The study focuses on experimentally determining parameters required for accurate detector modelling and interpretation of measured spectra. The following sections detail the measurement methods and results, providing a consistent dataset for evaluating the performance of ZnSe(Al,O) in beta spectroscopy applications.

## 2. Methodology

**Figure 1:**
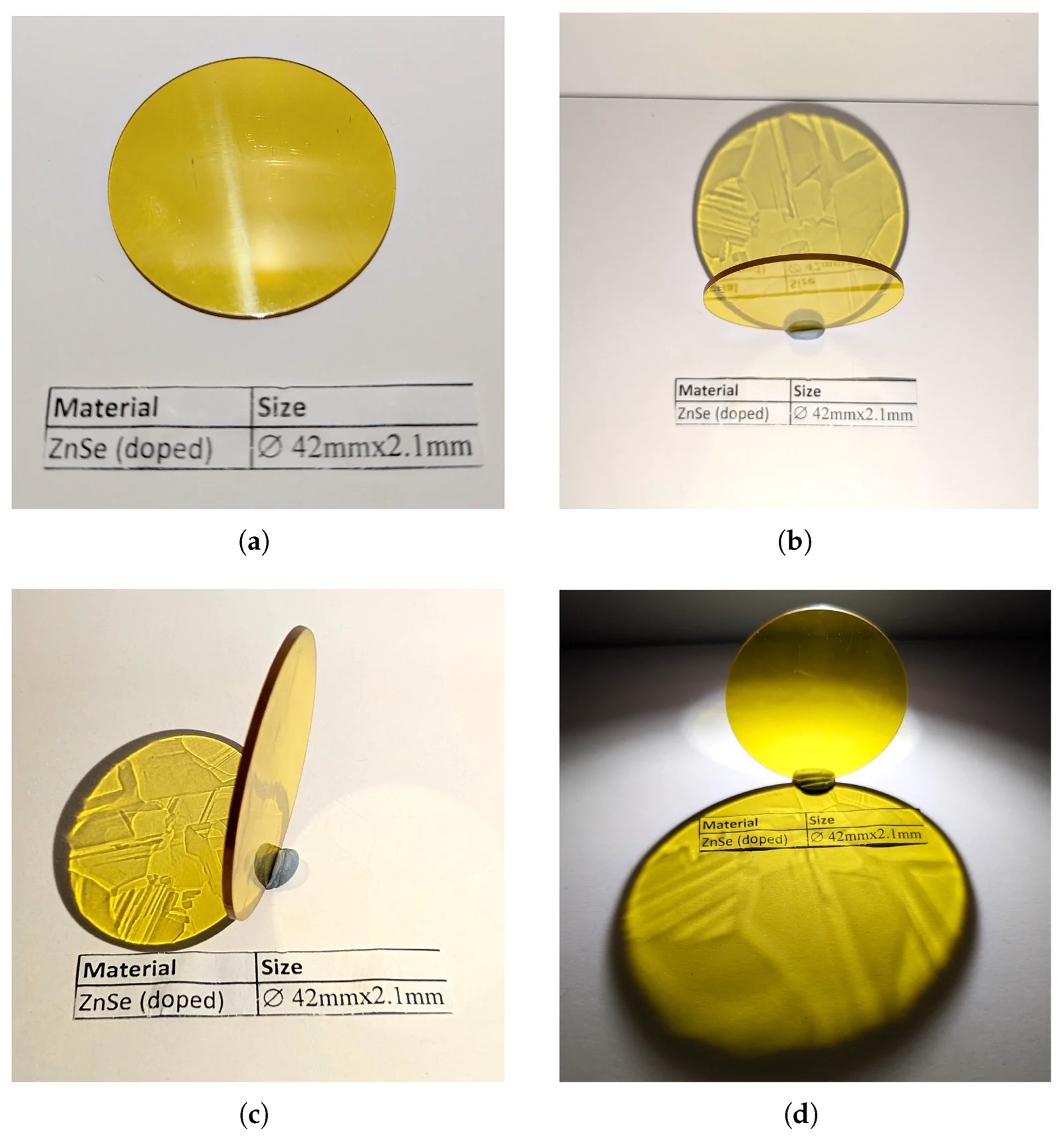
Photographs of the ZnSe(Al,O) crystal from different orientations, illustrating surface quality, geometry, and internal features revealed under transmitted illumination. A label beneath the crystal indicates the dimensions provided by the supplier. (**a**) Top view of the ZnSe(Al,O) crystal showing surface finish. (**b**) View of the sample from above showing transmitted-light image which highlights the growth lines. (**c**) Side view of the crystal illustrating thickness and edge quality. (**d**) Back-illuminated front view.

Sample characterisation is done on a ZnSe (doped) crystal provided by Advatech UK [12]. The material was intended to be co-doped with aluminium and oxygen; however, the data sheet provided by the supplier identified the sample as ZnSe(doped). In order to confirm the crystal’s properties and parameters, an independent study determining them was necessary. The crystal’s diameter is 42 mm while its thickness is 2.1 mm. The crystal’s faces appear to be highly polished but there are inclusions and growth defects which can be seen with the unaided eye. Figure 1 shows photos of the sample from different angles. When an external light source was shone through the crystal, the transmitted-light image clearly enhanced the contrast of the internal growth planes.

**Figure 2:**
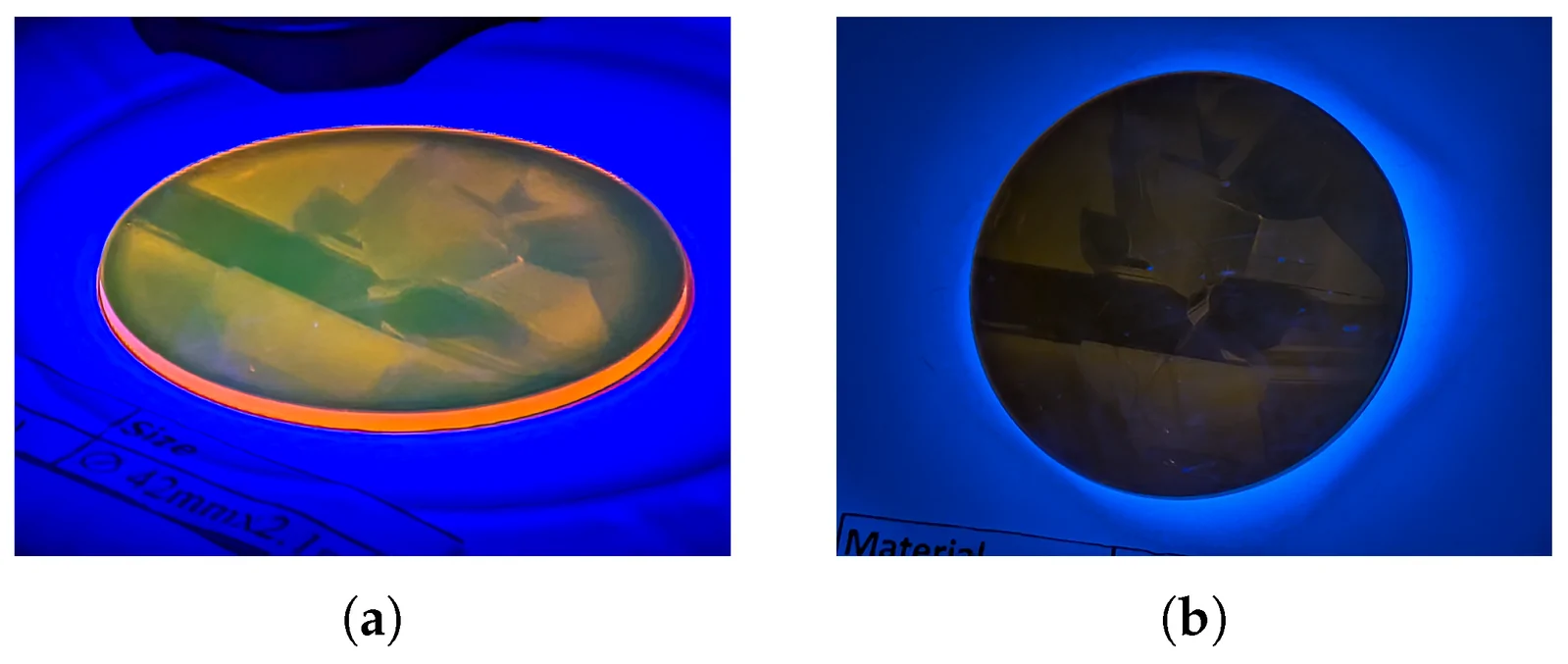
UV images of the ZnSe(Al,O) crystal illustrating variations in optical response and internal structure under ultraviolet excitation. (**a**) UV illumination of the ZnSe(Al,O) crystal showing surface and internal features. (**b**) Alternate UV illumination highlighting variations in optical response.

Figure 2 shows the crystal under UV illumination, where the internal growth features are much more visible. The increased contrast comes from variations in how the crystal transmits and interacts with light across its structure. ZnSe crystals are notoriously difficult to grow as a single crystal due to its tendency for twinning. Twinning in ZnSe happens due to its high dissociation at melting point that destroys single crystal structure [14].

### 2.1. Chemical Composition Analysis

Since the dopant composition was not specified, the crystal was analysed using a scanning electron microscope (SEM) equipped with energy-dispersive X-ray spectroscopy (EDS). To minimise charging effects during imaging, a conductive carbon layer was applied to the sample surface. The analysis was primarily aimed at determining whether tellurium was present, given that ZnSe(Te) is the most common ZnSe-based scintillator on the market. The EDS measurements were used to identify the constituent elements and estimate their relative atomic abundances. The technique was also used to image the crystal and investigate the composition of the observed artefacts through elemental mapping. Because EDS provides only semi-quantitative compositional information, particularly for the lower-abundance Al and O signals, these measurements were not treated as a high-precision determination of bulk stoichiometry.

### 2.2. Density Measurement

The density of the crystal was determined by first weighing the crystal using precise scales, and then measuring the radius and thickness of the crystal with a digital calliper. The density was then calculated as

(1) $$\rho =\frac{m}{\pi r^{2}h},$$

where ρ is the density and r and h are the radius and thickness of the crystal, respectively.

### 2.3. Refractive Index Measurement

**Figure 3:**
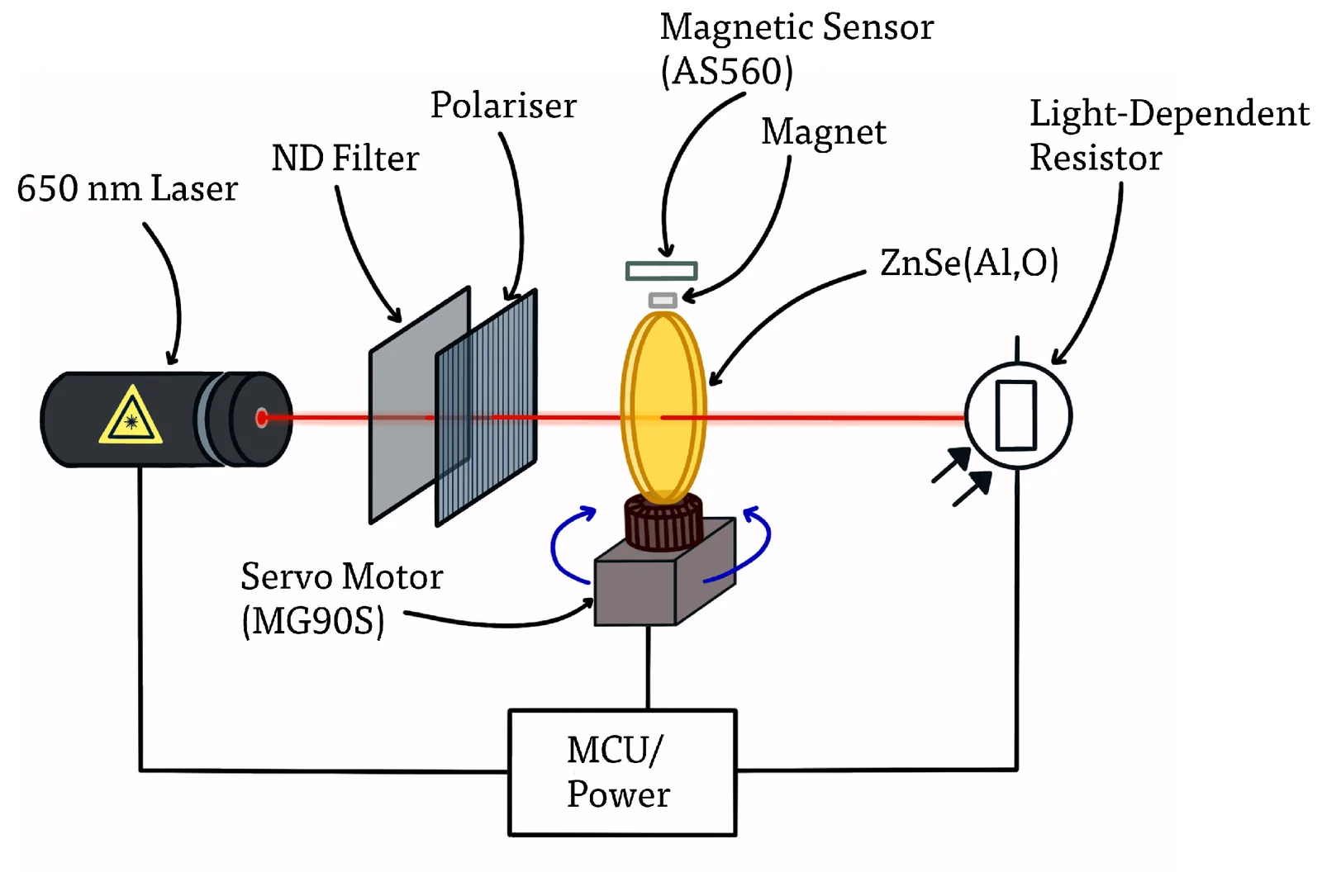
Experimental setup used to determine the refractive index of the ZnSe(Al,O) crystal by measurement of the Brewster angle. A 650 nm laser was passed through an ND filter and linear polariser before being directed through the crystal, which was mounted on an MG90S servo motor. The transmitted light intensity was measured using a light-dependent resistor (LDR), while the servo position and LDR response were controlled and recorded using an Arduino Due microcontroller. ND filters were needed to ensure that the transmitted light intensity fell within the dynamic range of the LDR.

The refractive index of the ZnSe(Al,O) crystal was determined from its Brewster angle using an automated transmitted-light measurement. A 650 nm laser was directed through a neutral density (ND) filter and a linear polariser to produce an attenuated, p-polarised beam. The beam was aligned with the crystal’s central axis. The crystal was mounted vertically on an MG90S servo motor (TowerPro, Singapore, unit sourced from China), as shown in Figure 3.

The servo rotated the crystal through an angular range of $90^{\circ }$, varying the angle of incidence of the laser beam. A light-dependent resistor (LDR) was positioned behind the crystal and aligned with the transmitted beam. The LDR continuously measured the transmitted light intensity. An Arduino Due microcontroller (MCU) controlled the angular position of the servo and simultaneously recorded the corresponding LDR response. The measured angle and light-intensity data were exported as a CSV file and analysed to obtain the transmitted intensity as a function of incidence angle.

Initially, the angular position was determined from the commanded servo position, with a nominal angular resolution of $1^{\circ }$. Twenty complete measurement cycles were recorded using this configuration. The angular measurement was subsequently refined by mounting a magnet above the crystal, coaxial with the servo rotation axis, and positioning an AS5600 magnetic rotary-position sensor (ams OSRAM, Munich, Germany, unit sourced from China) approximately 6 mm above the magnet. The sensor provided an angular resolution of approximately 0.0878°, allowing the actual angular position of the crystal to be measured independently of the commanded servo position. A further five complete measurement cycles were recorded using this configuration.

The Brewster angle, $\theta_{B}$, was identified as the angle at which the transmitted intensity of the p-polarised beam reached a maximum, corresponding to the minimum in reflected intensity at the air–crystal interface. The refractive index was then determined using the Brewster relation,

(2) $$\tan\theta_{B}=\frac{n_{\mathrm{ZnSe}(\mathrm{Al},O)}}{n_{\mathrm{air}}},$$

where $\theta_{B}$ is measured relative to the surface normal. Taking $n_{\mathrm{air}}\approx 1$, the refractive index of the crystal was therefore calculated as

(3) $$n_{\mathrm{ZnSe}(\mathrm{Al},O)}=\tan\theta_{B}.$$

### 2.4. Light Yield Determination

**Figure 4:**
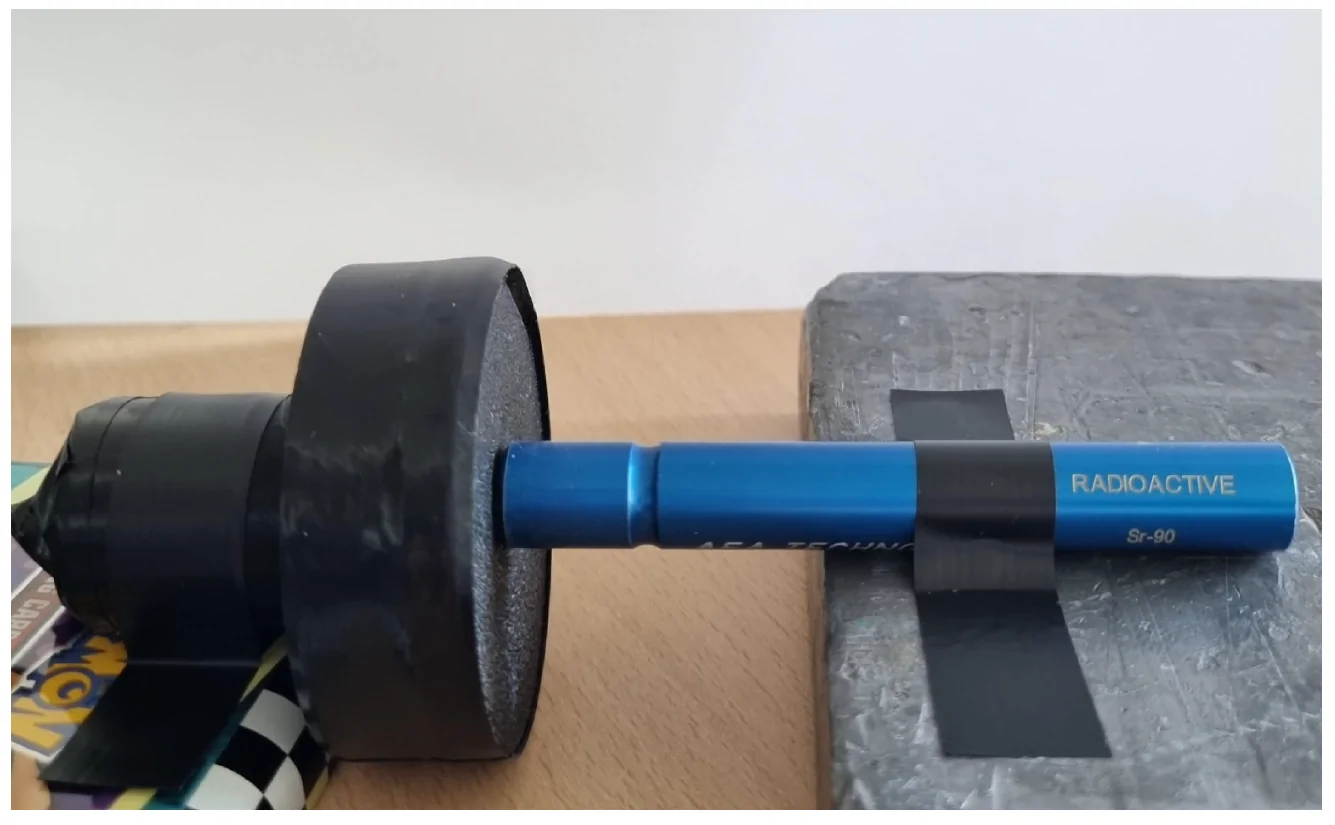
Experimental setup showing the Sr-90 source aligned with the detector assembly for beta irradiation measurements.

**Figure 5:**
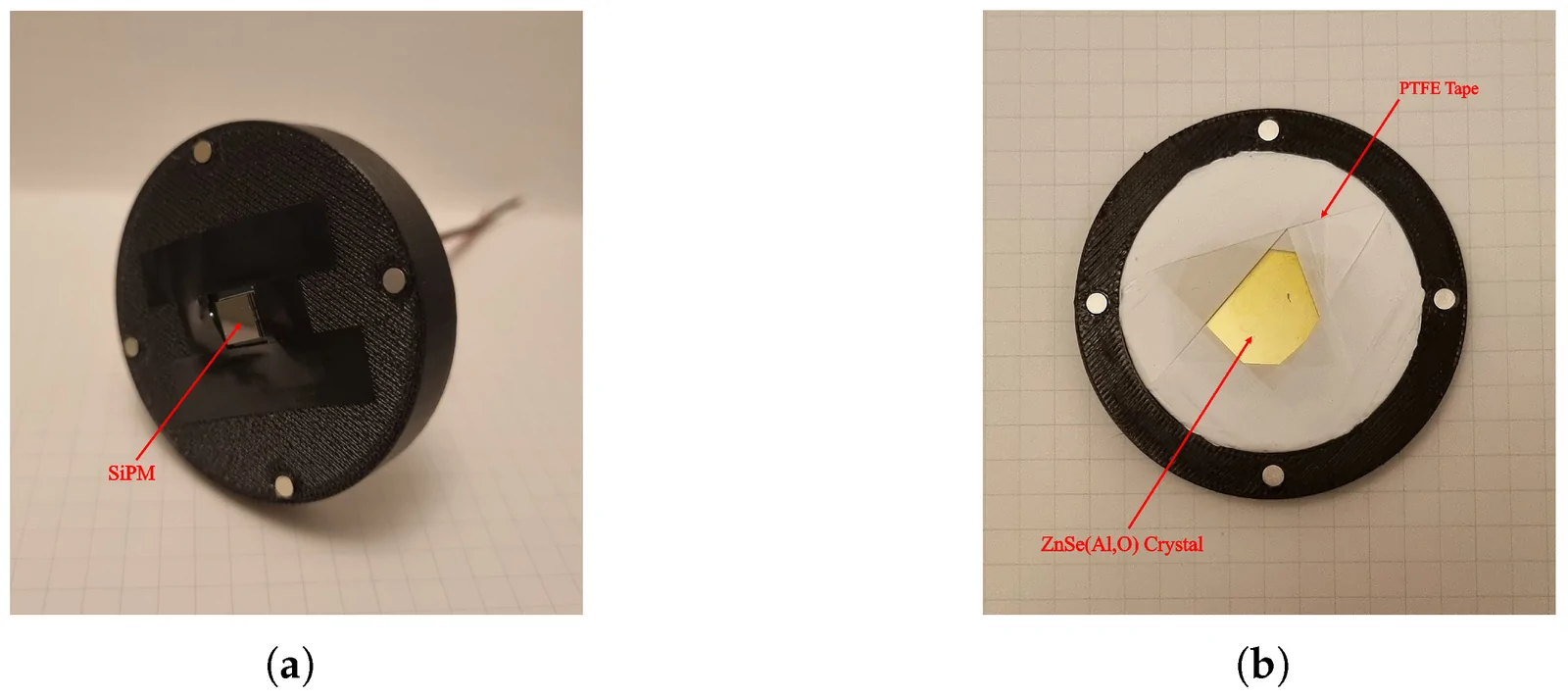
Detector components showing (**a**) the centrally positioned SiPM and (**b**) the ZnSe(Al,O) crystal assembly, illustrating the alignment between the sensor and scintillator within the housing. (**a**) SiPM assembly within the detector housing, showing the position of the sensor at the centre of the enclosure—which aligns with the uncovered part of the crystal in (**b**). (**b**) ZnSe(Al,O) crystal mounted within the detector housing, with PTFE wrapping used to enhance light collection.

To determine the intrinsic light yield of the ZnSe(Al,O) scintillator from digitised waveforms, an automated analysis pipeline was developed in Python (v3.12.3). Prior to this, data was collected using the setup shown in Figure 4. A Sr-90 source was placed in front of the detector and 10,000 pulses were recorded. The detector consisted of the ZnSe(Al,O) crystal coupled to an SiPM housed in a 3D printed case (shown in Figure 5). Two sets of data were taken; one with the crystal coupled to a Broadcom AFBR-S4N66P014M SiPM (Broadcom Inc. Palo Alto, CA, USA, unit sourced from the UK.) and another set coupled to a Hamamatsu S14420-3050MG (Hamamatsu Photonics, Hamamatsu City, Japan, unit sourced the UK.). This allowed for two independent measurements of the same observable. Signals from the detector were processed by a custom readout circuit. The pipeline converts electrical pulse data into physical observables through four stages: data preprocessing, charge integration, endpoint determination, and calibration.

The pipeline processes individual pulse waveforms recorded as voltage-time series. A baseline correction is applied to each event to remove DC offsets and low-frequency noise. This is done by calculating the mean of a pre-trigger region (e.g., the first 50 samples) and subtracting it from the full waveform. The pulse peak is then identified, and an integration window is defined to isolate the signal region. The total charge of each pulse is obtained by integrating the voltage signal using the trapezoidal rule. The integrated area, in Volt-seconds (V·s), is converted to charge using the system resistance (*R*) and amplifier gain ($G_{\mathrm{amp}}$):

(4) $$Q_{\mathrm{event}}=\frac{\int V(t)\;dt}{R\times G_{\mathrm{amp}}}$$

The resulting charge values are used to construct a charge spectrum corresponding to the deposited beta energies. The Sr-90/Y-90 beta spectrum is continuous, with a maximum energy of 2.28MeV from Sr-90/Y-90. To determine the endpoint, the charge spectrum is binned into a histogram and smoothed using a Gaussian filter to reduce statistical fluctuations. The Sr-90/Y-90 beta spectrum is continuous, with a maximum energy of 2.28 MeV from Y-90. To determine the endpoint, the charge spectrum was binned into a histogram and smoothed using a Gaussian filter to reduce statistical fluctuations. A linear fit was then applied to the high-charge tail of the smoothed spectrum. The endpoint was determined from the extrapolated intercept of this fit, providing a consistent estimate of the maximum detected charge while reducing sensitivity to statistical fluctuations in the tail. The resulting endpoint charge, $Q_{end}$, was then used to estimate the intrinsic light yield. This provides a consistent estimate of the maximum energy while suppressing outliers such as pile-up events. The charge corresponding to the endpoint ($Q_{\mathrm{end}}$) is used to estimate the intrinsic light yield. The charge produced by a single photoelectron is given by:

(5) $$Q_{1\mathrm{pe}}={\mathrm{Gain}}_{\mathrm{SiPM}}\times e\times \left(1+P_{\mathrm{xtalk}}\right)$$

The total number of photoelectrons is then:

$$N_{\mathrm{pe}}=\frac{Q_{\mathrm{end}}}{Q_{1\mathrm{pe}}}$$

The detected yield is obtained by normalising to the endpoint energy:

$$\frac{N_{\mathrm{pe}}}{2.28\;\mathrm{MeV}}$$

The detected yield is corrected for both the photon detection efficiency (PDE) of the SiPM and the optical collection efficiency (CE). The CE accounts for geometric mismatches and optical losses, most notably total internal reflection at the ZnSe interface. This procedure converts the measured electrical signal into an estimate of the intrinsic number of photons produced per unit of deposited energy. While the PDE was sourced from the respective SiPM data sheets, the CE was estimated for the different photodetector geometries using a Geant4 Monte Carlo optical simulation. The simulation explicitly modelled the scintillator volume, the diffuse PTFE wrapping, and the optical grease layer coupling the crystal to the SiPM. By generating and transporting photons through these simulated geometries, an accurate estimate of the collection efficiency for each assembly was obtained.

### 2.5. Decay Time Analysis

A ZnSe(Al,O) crystal was optically coupled to a Hamamatsu S14420-3050MG SiPM using optical grease and read out with an oscilloscope through custom biassing and readout circuitry. A total of 10,000 scintillation pulses were recorded and analysed using Python.

The falling edge of each waveform was fitted using the single-exponential function given in Equation (6),

(6) $$A\left(t\right)=A_{0}e^{-\frac{t}{\tau }}+C$$

where A(t) is the pulse amplitude at time *t*, $A_{0}$ is the amplitude at the start of the decay, τ is the decay time constant, and *C* is a constant baseline term. The fit window began five samples after the pulse maximum and extended to the end of the recorded waveform, excluding the pulse-rise region. Fits were retained only where $R^{2}\geq 0.90$ and the fitted uncertainty in τ was less than 15% of the fitted value. These criteria were used to reduce the influence of noisy or poorly fitted events. The mean decay constant was then calculated from the accepted waveform population, together with the waveform-to-waveform standard deviation and the standard error on the mean.

### 2.6. Emission Spectroscopy

Scintillators emit visible light following excitation. The emission spectrum is strongly influenced by the crystal structure, defect states, and dopant composition. Characterising the emission spectrum provides insight into the optical behaviour of the scintillator and its compatibility with photo-sensors such as SiPMs.

In this work, the emission spectrum of the ZnSe(Al,O) crystal was measured using a Raspberry Pi-based spectrometer. The crystal was positioned at the spectrometer aperture such that the centre of the sample was aligned with the optical axis. A UV torch, with wavelength 365 nm, was then used to excite the crystal, producing photo-luminescence which was collected and dispersed by the spectrometer monochromator onto a CCD sensor. The recorded intensity as a function of wavelength was then used to produce the characteristic emission spectrum of the scintillator.

## 3. Results & Discussion

### 3.1. Chemical Composition Analysis

**Figure 6:**
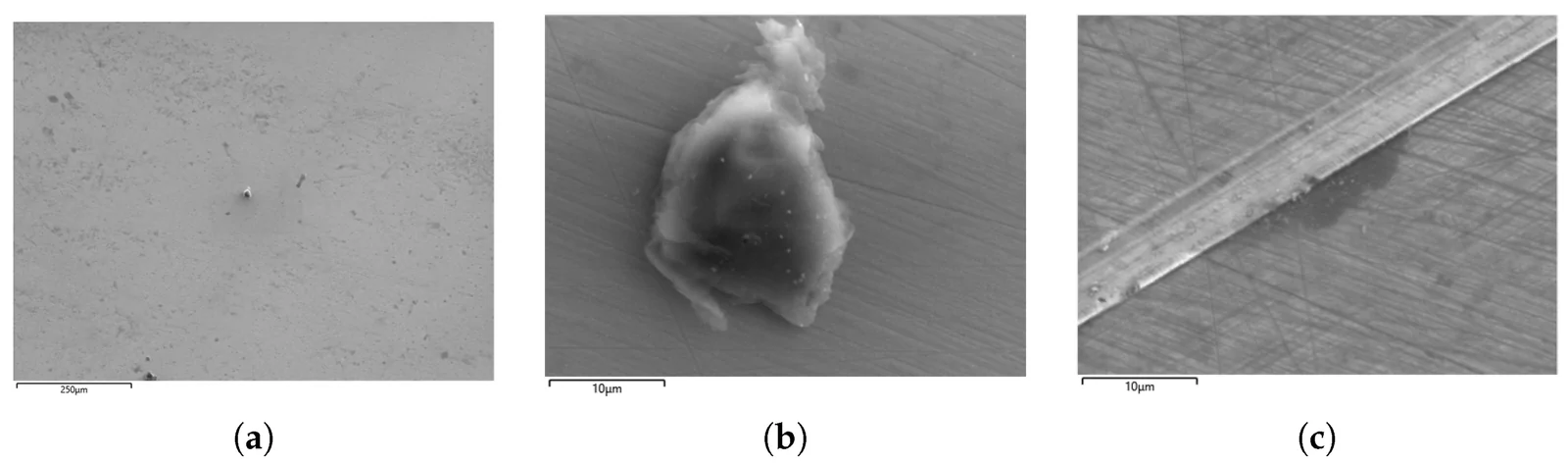
SEM images of the ZnSe(Al,O) crystal showing surface morphology and representative localised defects at different magnifications. (**a**) SEM image of the crystal surface at 250× magnification. (**b**) SEM image of a representative surface artefact at 5000× magnification. (**c**) SEM image of a representative surface artefact at 5000× magnification.

**Figure 7:**
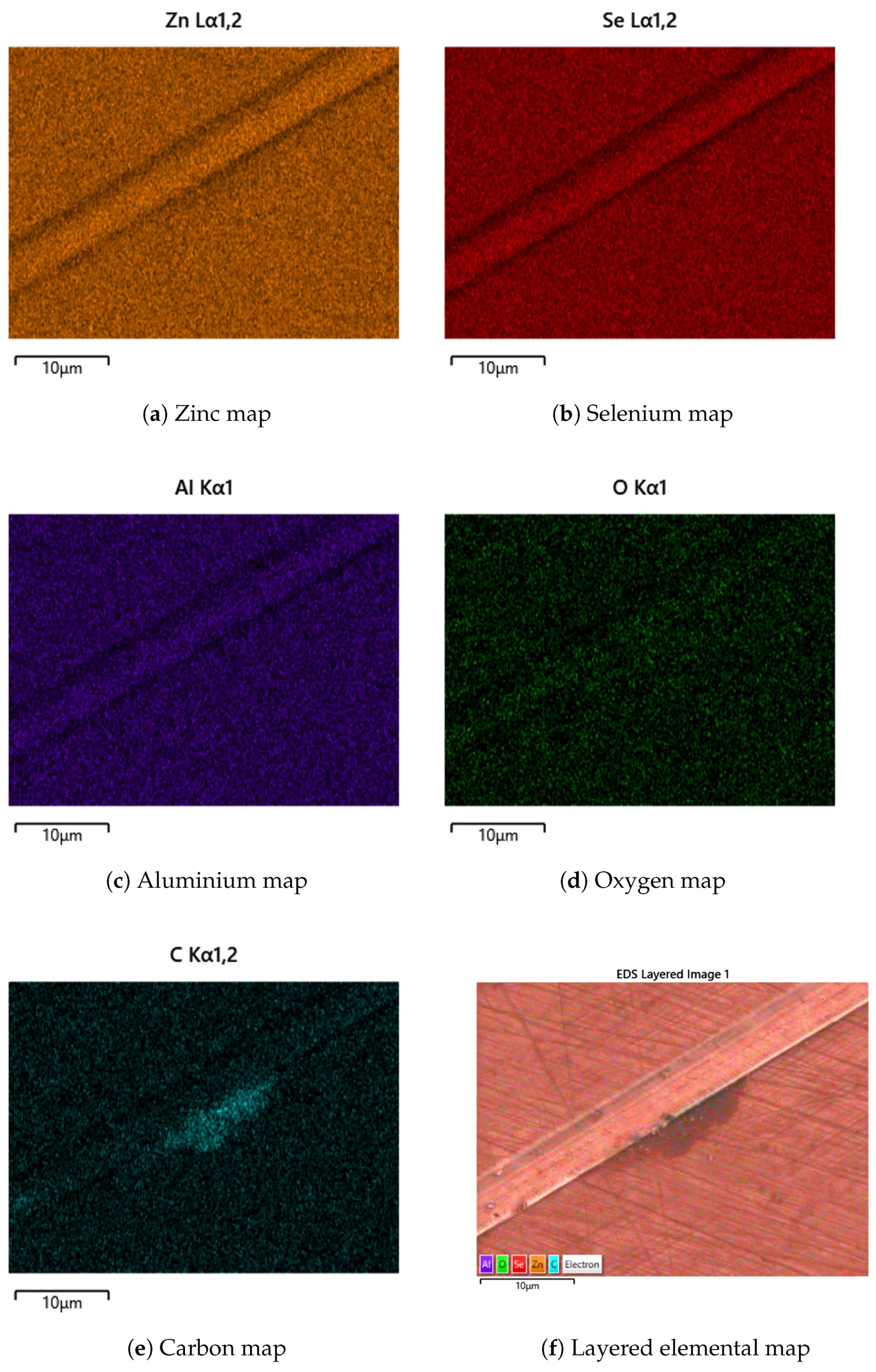
SEM-EDS elemental maps of the ZnSe(Al,O) crystal showing the spatial distribution of Zn, Se, Al, O, and C across the observed surface artefact. The linear feature is visible across the Zn, Se, and Al maps, while the carbon map indicates localised surface contamination. The analysed region corresponds to the feature shown in Figure 6c.

The SEM analysis began with imaging of the crystal surface, shown in Figure 6. At low magnification, the surface appears relatively uniform; however, several macroscopic artefacts are visible to the unaided eye. These features were investigated in more detail by increasing the magnification, with representative examples shown in Figure 6b,c. The artefacts exhibit clear structural irregularities, indicating local variations in crystal growth and material quality. To further investigate the origin of these features, SEM-EDS elemental mapping was performed across the artefact region, as shown in Figure 7. The Zn, Se, and Al maps show the linear feature across the analysed region without significant elemental segregation, while the oxygen map exhibits comparatively weak contrast due to the low oxygen signal intensity. A localised enhancement is observed in the carbon map, indicating that part of the feature is associated with surface contamination rather than a major compositional variation within the crystal bulk. The broadly consistent elemental distribution across the remaining maps suggests that the observed artefacts are more likely associated with crystal growth irregularities and surface features than large-scale elemental inhomogeneity.

**Figure 8:**
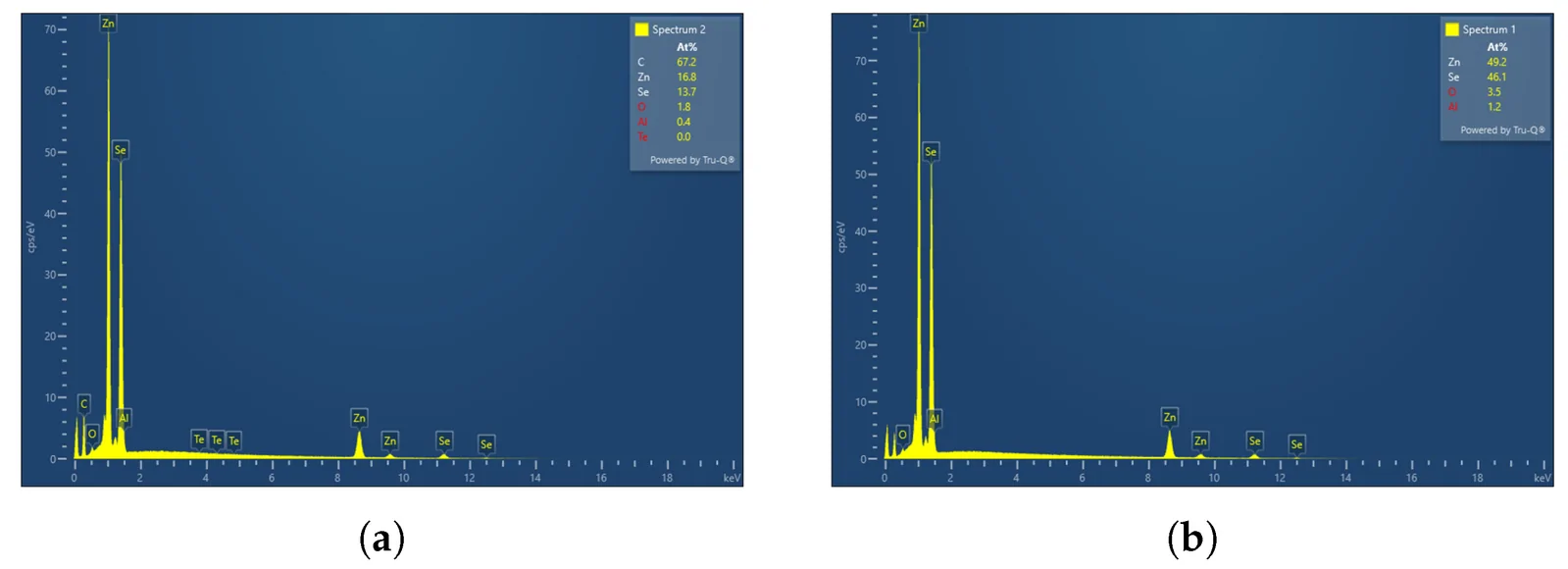
SEM-EDS spectra of the ZnSe(Al,O) crystal showing the measured elemental composition before and after excluding contaminant and trace-element contributions. Carbon was attributed to surface contamination. (**a**) SEM-EDS spectrum of the ZnSe(Al,O) crystal showing all analysed elements, including carbon and tellurium, which were intentionally included in the analysis to assess their presence within the crystal. (**b**) SEM-EDS spectrum showing the elemental composition of the ZnSe(Al,O) crystal after excluding carbon and tellurium from the elemental analysis.

To assess the elemental composition, EDS spectra were collected from regions of the crystal surface that did not contain prominent artefacts. The raw spectrum, shown in Figure 8a, contains a significant carbon peak, which is attributed to surface contamination and the conductive carbon coating applied prior to SEM analysis. After subtracting the carbon contribution and normalising the remaining signal, the underlying elemental distribution of the crystal is obtained. An additional spectrum, shown in Figure 8b, was analysed with the carbon contribution excluded. The resulting atomic abundance percentages were averaged with the carbon-subtracted values obtained from Figure 8a. The averaged elemental abundances are presented in Table 2.

The resulting composition is consistent with a ZnSe-based material with additional oxygen and aluminium content, supporting the expected ZnSe(Al,O) formulation. Approximate elemental composition estimated from SEM-EDS analysis: ${\mathrm{ZnSe}}_{0.875}O_{0.089}{\mathrm{Al}}_{0.024}$.

The averaged EDS atomic abundances were normalised to Zn to provide the approximate compositional ratio ZnSe_0.875_O_0.089_Al_0.024_. This representation is derived directly from the measured relative atomic abundances and is not intended as an independently determined crystallographic stoichiometry. The averaged Se:Zn ratio was 0.875, compared with the ideal 1:1 ratio of stoichiometric ZnSe. Both individual EDS measurements also gave Se:Zn ratios below unity, at approximately 0.815 and 0.937, indicating a consistent relative Se deficiency across the analysed regions.

### 3.2. Refractive Index Measurement

**Figure 9:**
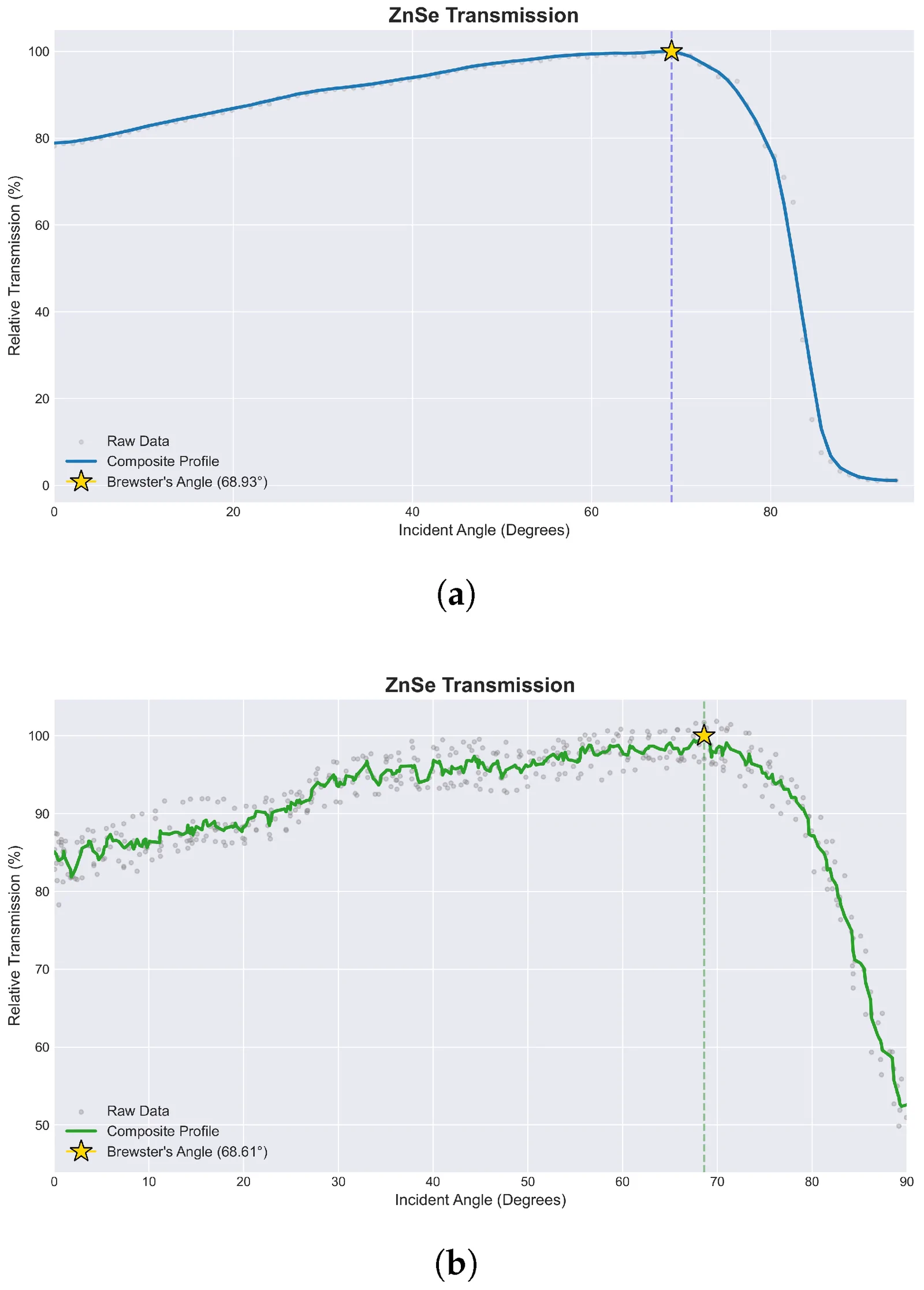
Normalised transmitted-light intensity of the 650 nm p-polarised laser as a function of incidence angle for the ZnSe(Al,O) crystal. The transmitted intensity was normalised to the maximum recorded value for each measurement. Both angular measurement methods produced a clear transmission maximum at approximately $69^{\circ }$, corresponding to the Brewster angle of the crystal. (**a**) Normalised transmission profile obtained using the commanded MG90S servo position, giving a Brewster angle of 68.93°. (**b**) Normalised transmission profile obtained using the AS5600 magnetic position sensor, giving a Brewster angle of 68.61°.

Figure 9 shows the normalised transmitted intensity as a function of incident angle for the ZnSe(Al,O) crystal. Both configurations, using the servo alone and the servo with the magnetic sensor, produce the same characteristic profile. The transmitted intensity increases with incident angle and reaches a maximum at approximately $69^{\circ }$, corresponding to the Brewster angle, before decreasing again at larger angles. Brewster-angle behaviour is commonly illustrated in terms of reflectance [15]. Since, for an ideal non-absorbing interface, T=1−R, the transmission profile is the inverse of the corresponding reflectance curve. The measured profiles in Figure 9 therefore show the expected angular dependence.

Using the servo position alone, the maximum transmitted intensity occurred at 68.93°, corresponding to a refractive index of 2.596. With the AS5600 magnetic position sensor, the maximum was measured at 68.61°, corresponding to a refractive index of 2.553. Together, the two refractive indices measured give an average of 2.574. They also differ by only 0.32° showing good agreement between the two methods. This value also agrees with the refractive indices shown in Table 1, and in [16] which reports a refractive index of 2.568 at 650 nm.

The Fresnel reflectance at normal incidence is given by

(7) $$R={\left(\frac{n_{1}-n_{2}}{n_{1}+n_{2}}\right)}^{2}.$$

Using $n_{1}=1$ for air and $n_{2}=2.596$ gives R≈0.198, corresponding to approximately 20% reflection at a single air–ZnSe interface. The measured transmission at $0^{\circ }$ is approximately 80% of its maximum value, which is qualitatively consistent with the reduction in transmission expected from Fresnel reflection. However, the plotted values represent transmission relative to the measured maximum rather than absolute optical transmittance.

### 3.3. Light Yield Determination

**Figure 10:**
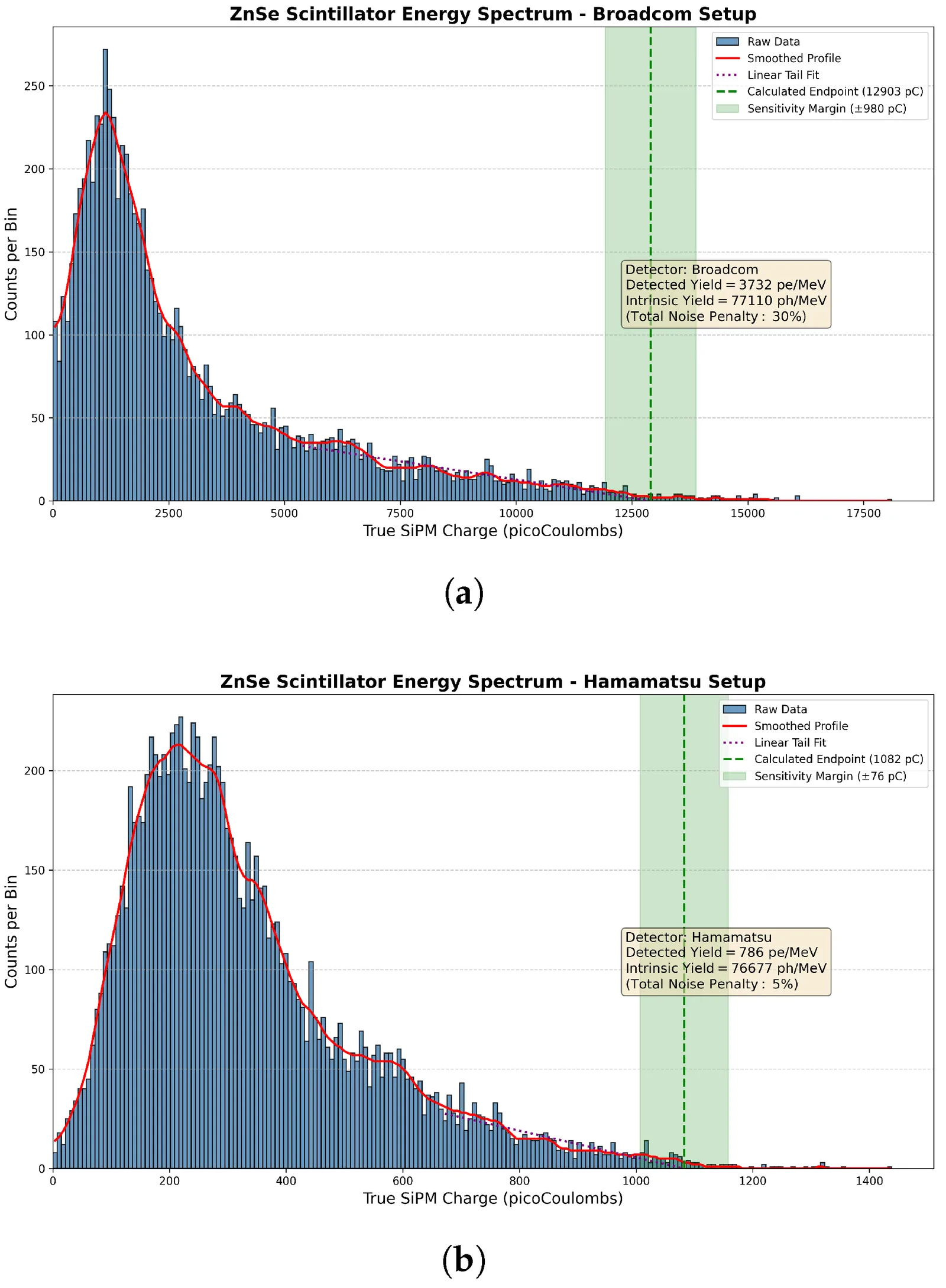
Charge spectra obtained from the ZnSe(Al,O) detector using two SiPM models. The spectra were used to estimate the detected charge endpoint for light-yield calculation. (**a**) Charge spectrum measured using the Broadcom SiPM. (**b**) Charge spectrum measured using the Hamamatsu SiPM.

The charge spectra obtained using the Broadcom and Hamamatsu SiPMs are shown in Figure 10. In both cases, the spectra exhibit the characteristic continuous distribution expected from Sr-90/Y-90 beta decay, with a high population of lower-charge events and a gradual fall-off towards the endpoint region. Gaussian smoothing was applied to suppress statistical fluctuations while preserving the overall spectral shape. A linear fit was then applied to the high-charge tail to determine the endpoint. The calculated charge endpoints were approximately 12,903 pC for the Broadcom SiPM and 1082 pC for the Hamamatsu SiPM. The shaded regions in Figure 10 show the sensitivity of the endpoint determination to the fitting procedure, corresponding to approximately ±980 pC and ±76 pC for the Broadcom and Hamamatsu measurements, respectively.

The substantial difference in absolute charge scale arises from the different detector characteristics of the two SiPMs. In particular, the Broadcom device has a considerably larger active area and higher optical collection efficiency than the Hamamatsu device, resulting in a much larger detected photoelectron yield. Differences in SiPM gain, PDE and crosstalk also contribute to the measured charge response.

After correcting for SiPM gain, photon detection efficiency (PDE), and optical collection efficiency (CE), the estimated intrinsic light yields obtained from both systems were in strong agreement, with values of approximately 77,110 photons/MeV and 76,677 photons/MeV for the Broadcom and Hamamatsu configurations, respectively. Table 3 summarises the detector-specific parameters used in these corrections together with the corresponding detected and corrected light-yield values. The close agreement between the two independently obtained values supports the consistency of the light-yield extraction method. The final reported value in Table 4 was taken as the average of the two measurements.

The spectra also demonstrate the relatively broad detector response expected from beta interactions in a scintillator detector system. Unlike mono-energetic gamma interactions, beta particles deposit energy continuously throughout the crystal volume, producing a wide range of scintillation intensities. Additional spectral broadening is likely introduced by photon transport losses within the crystal, electronic noise, SiPM statistical gain fluctuations, and event-to-event variations in optical coupling and light collection.

A noticeable low-charge enhancement is present in both spectra, particularly in the Broadcom measurement. This region is likely influenced by partial-energy deposition events, electronic noise close to threshold, and lower-energy beta interactions. At higher charges, the spectra gradually approach the endpoint without a sharp cut-off, reflecting both the continuous Sr-90/Y-90 beta spectrum and the finite energy resolution of the detector system.

The observed intrinsic light yield of $7.69\times 10^{4}$ photons ${\mathrm{MeV}}^{-1}$ is within the range reported for ZnSe-based scintillators, but is higher than the values previously quoted for ZnSe(Al,O), which are typically given as up to $5\times 10^{4}$ photons ${\mathrm{MeV}}^{-1}$ [4,10]. In these earlier studies, the ZnSe(Al,O) value is presented as a tabulated material property rather than being derived through a clearly described absolute light-yield extraction procedure for that specific material. Cho et al. also note that absolute light-output measurements are experimentally complex, and their own measurements focus mainly on comparative pulse-height spectra for ZnSe(Te), ZnSe(O), and CsI(Tl) detector configurations [4]. The value reported here therefore provides a directly measured light yield for the specific ZnSe(Al,O) crystal used in this detector system, obtained through a reproducible waveform-integration and Geant4-validated SiPM-correction procedure.

Furthermore, from a physio-chemical perspective, this enhanced light yield reflects modern advancements in crystal growth that minimise non-radiative recombination pathways [17]. Historically, the light yield of ZnSe scintillators was limited by deep-level trap states associated with intrinsic point defects, such as zinc vacancies, and unintentional impurities [18,19]. Highly optimised aluminium and oxygen co-doping, combined with improved stoichiometric purity, significantly reduces the density of these non-radiative defect centres [20]. This allows modern materials to push past historical limitations and closer toward the theoretical limits dictated by the host lattice bandgap. This performance is entirely consistent with modern state-of-the-art material quality; commercial manufacturers of specialised ZnSe(Al,O) scintillators now routinely specify nominal light yields of $7\times 10^{4}$ photons ${\mathrm{MeV}}^{-1}$ [21]. Therefore, the measured yield places the investigated crystal firmly within the expected performance bounds of a highly optimised, high-purity ZnSe(Al,O) sample.

**Table 3:** Detector-specific parameters used in the light-yield calculation and the resulting detected and corrected light yields for the Broadcom AFBR-S4N66P014M and Hamamatsu S14420-3050MG SiPMs.

Parameter	Broadcom AFBR-S4N66P014M	Hamamatsu S14420-3050MG
Active area (${\mathrm{mm}}^{2}$)	36 [22]	7.07 [23]
SiPM gain	$7.3\times 10^{6}$ [22]	$3.6\times 10^{6}$ [23]
PDE at ZnSe(Al,O) peak emission	41% [22]	39% [23]
Optical CE (Geant4-derived)	12.066%	3.027%
Detected yield (pe ${\mathrm{MeV}}^{-1}$)	3732	786
Corrected light yield (ph ${\mathrm{MeV}}^{-1}$)	77,110	76,677

**Table 4:** Experimentally measured and literature-quoted material and scintillation properties of the ZnSe(Al,O) crystal relevant to detector modelling.

	ZnSe(Al,O) (Experimental)	ZnSe(Al,O) (Literature)
Dopants	Al, O	Al, O
Elemental composition (%)	Zn: 50.3, Se: 44.0, Al: 1.2, O: 4.5	0.03–0.1 [6]
Density (${\mathrm{g cm}}^{-3}$)	5.31	
Refractive index	2.574	2.568 [16]
Light yield (${\mathrm{photons MeV}}^{-1}$)	76 894	5$\times 10^{4}$ [4,10]
Decay time (μs)	9.41 ^10^	3–15 [4,10]
Peak emission wavelength (nm)	581	575–650 [4,6]

^10^ The waveform-to-waveform standard deviation was 1.1 μs and the standard error on the mean was 0.01 μs. The standard error reflects the precision of the mean and is not treated as the total experimental uncertainty.

The detected yields differed by a factor of 4.75, with 3732 pe/MeV measured using the Broadcom SiPM and 786 pe/MeV using the Hamamatsu SiPM. This difference is expected from the substantially different detector geometries. The Broadcom device has an active area of $36\;{\mathrm{mm}}^{2}$, compared with $7.07\;{\mathrm{mm}}^{2}$ for the Hamamatsu device, giving an active-area ratio of approximately 5.09. The independently determined Geant4 optical collection efficiencies were 12.066% and 3.029%, respectively, corresponding to a ratio of 3.98. When combined with the PDE ratio of 1.05, these detector-specific corrections predict a detected-yield ratio of approximately 4.19. This accounts for most of the measured 4.75-fold difference without deriving the correction factors from the measured light-yield data. The active-area ratio is not applied as an additional correction because the SiPM geometry is already included in the Geant4-derived collection efficiency.

### 3.4. Decay Time Analysis

The scintillation decay time of the ZnSe(Al,O) detector system was measured to be 9.41 μs, obtained from single-exponential fitting of the falling edge across the accepted waveform population. The waveform-to-waveform standard deviation was 1.1 μs, representing the spread in fitted decay constants between individual events. The standard error on the mean was 0.01 μs, reflecting the large number of accepted waveforms rather than the total experimental uncertainty. The measured value is therefore reported as an effective decay constant for the detector response.

**Figure 11:**
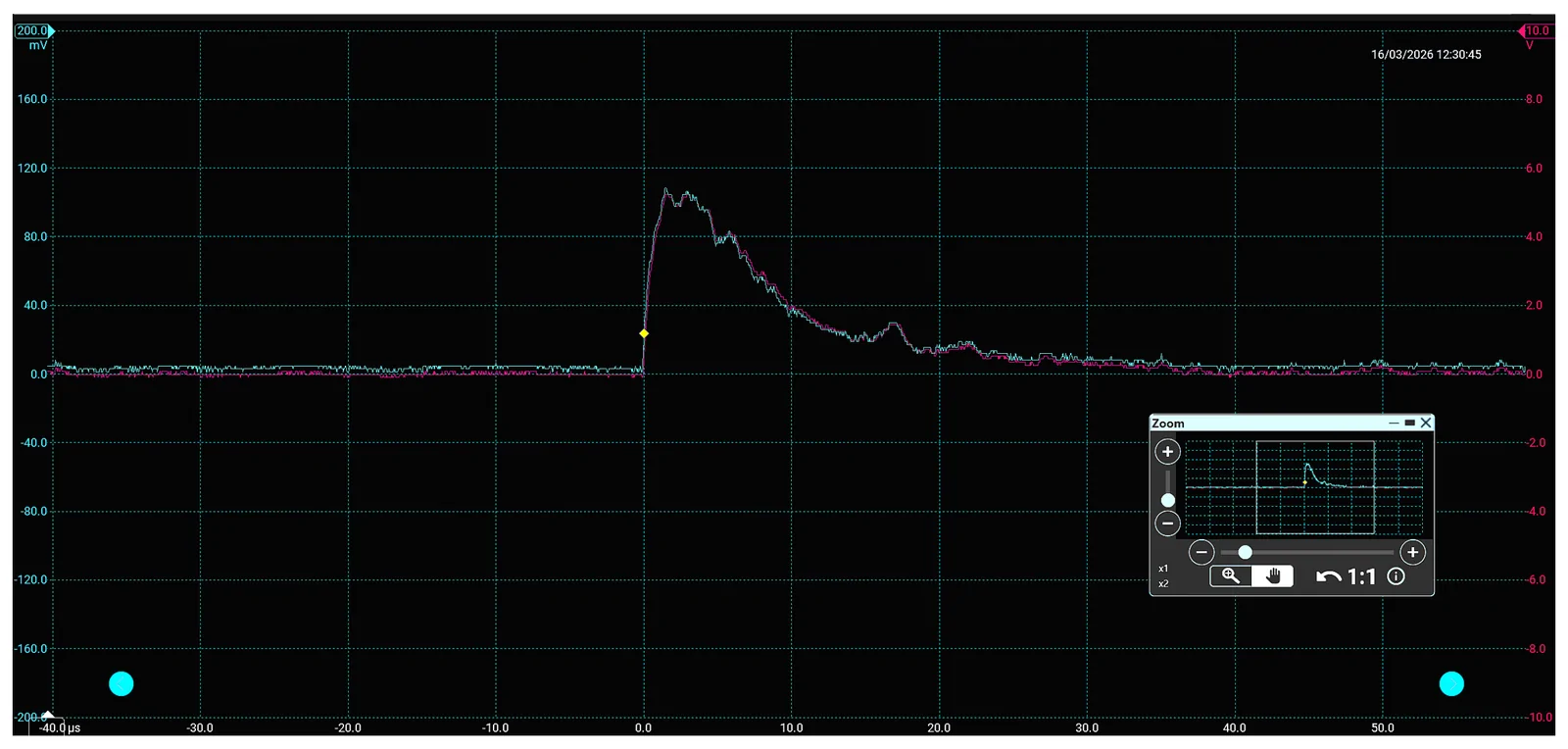
Full waveform pulse from detector with Hamamatsu SiPM. Bottom axis is time in μs. The left axis (blue) corresponds to the pre-amplified pulse response with a range up to 200 mV, while the right axis (red) shows the amplified pulse response with a range up to 10 V.

The measured pulse shapes exhibit the characteristic rapid rise and slower exponential decay expected from scintillation processes in ZnSe-based materials. A representative waveform obtained using the Hamamatsu SiPM configuration is shown in Figure 11.

The relatively slow decay measured in the case of the ZnSe(Al,O) single crystal corresponds well to other ZnSe-based scintillators reported in the literature, where radiative recombination via dopant and defect centres might result in microsecond decay components [8]. The relatively slower decay of scintillating materials can be favourable in beta spectroscopy, as it would mean more light pulses can be accumulated for better statistics in measuring the energy of detected photons. On the other hand, the presence of decay components in the order of microseconds would hinder performance in higher count rate systems due to pulse pile-up effects.

### 3.5. Emission Spectroscopy

**Figure 12:**
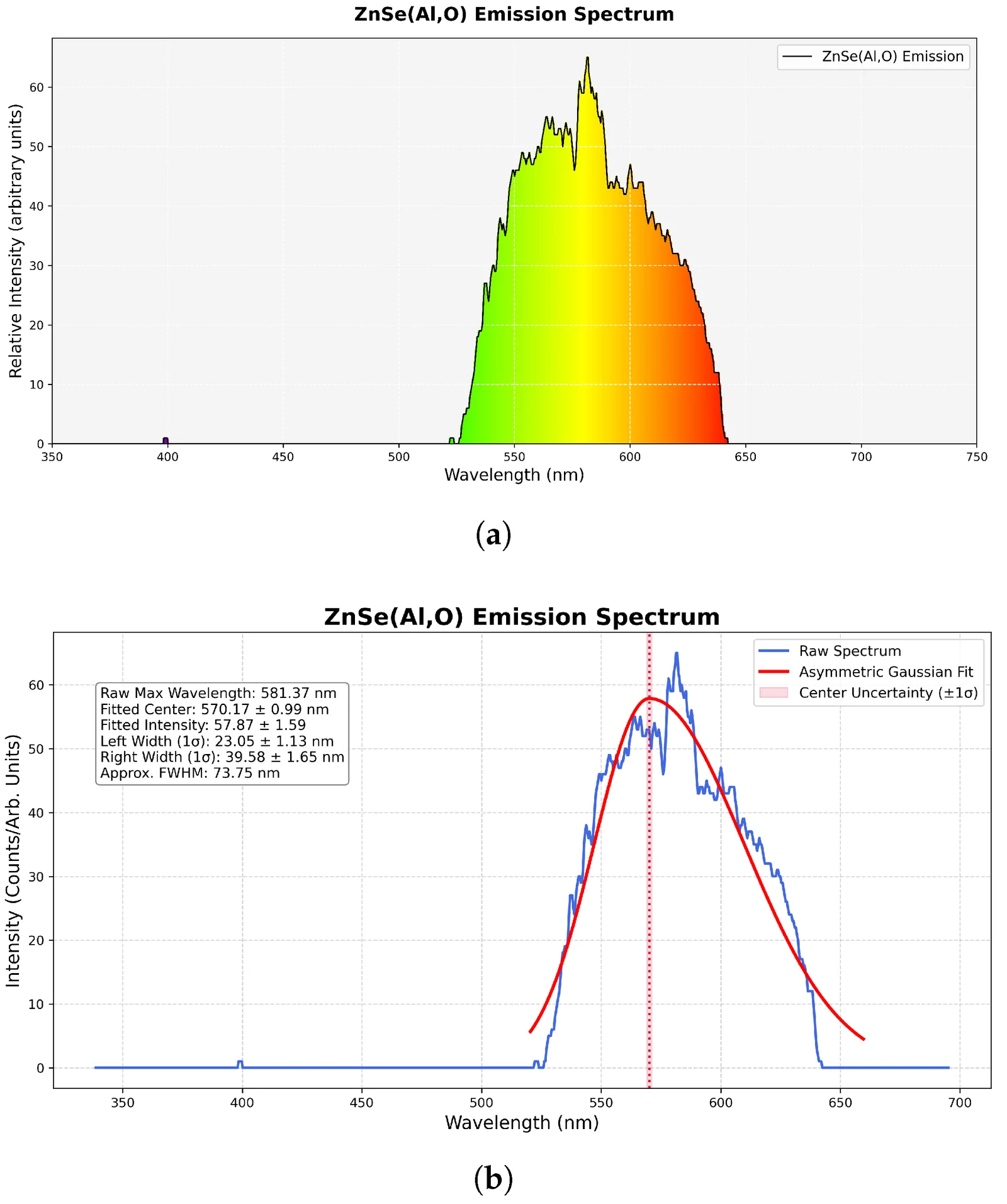
UV-excited emission spectra of the ZnSe(Al,O) crystal showing a broad visible emission band in the green-yellow to orange spectral region. The raw spectrum (blue) reaches maximum intensity at approximately 581 nm, while the asymmetric Gaussian fit (red) provides an empirical description of the non-symmetric emission profile. (**a**) UV-excited emission spectrum of the ZnSe(Al,O) crystal, showing a broad visible emission band with maximum intensity near 581 nm. (**b**) Emission spectrum of the ZnSe(Al,O) crystal with an asymmetric Gaussian fit applied to describe the broad, non-symmetric emission band.

The UV-excited (365 nm) emission spectrum of the ZnSe(Al,O) crystal is shown in Figure 12. The spectrum exhibits a broad visible emission band extending from approximately 522 nm to 642 nm, with the maximum measured intensity occurring at approximately 581 nm. An asymmetric Gaussian fit was applied to describe the non-symmetric emission profile, giving a fitted centre of 570.17 ± 0.99 nm and a FWHM of 73.75 nm. The fitted centre is therefore a parameter describing the asymmetric band and does not represent a second emission maximum. This result is consistent with reported emission behaviour for ZnSe-based scintillators, where Al-, O-, and Te-related defect states commonly produce broad yellow to orange-red emission bands rather than narrow line emission [6,8]. The peak emission wavelength is relevant to the detector design. A peak near 581 nm lies within the spectral response range of silicon photodetectors and SiPMs, meaning that the scintillation light produced by the crystal can be detected without the need for wavelength shifting. This supports the use of ZnSe(Al,O) in the current SiPM-coupled beta detector system. However, because many SiPMs have peak photon detection efficiency at shorter wavelengths, the measured emission spectrum should still be considered when selecting the most appropriate SiPM model for future detector optimisation.

Overall, the measured peak emission wavelength falls within the range reported for ZnSe(Al,O)-based scintillators, with previous studies reporting emission around 605 nm [4]. More specifically, the measured peak at 581 nm lies within the higher-energy emission band reported for ZnSe(Al,O), which has been observed between approximately 575 and 620 nm, rather than the lower-energy band reported around 640–650 nm [6].

## 4. Conclusions

The measured properties provide a consistent experimental parameter set for the ZnSe(Al,O) crystal used in the beta detector system. SEM-EDS confirmed the presence of Zn, Se, Al, and O and supported the expected co-doped ZnSe formulation. The measured density, refractive index, light yield, decay time, and emission wavelength were broadly consistent with values reported for ZnSe-based scintillators. Table 4 summarises the experimentally determined values and corresponding literature data.

The values reported in Table 4 should be interpreted as detector-relevant parameters for this specific crystal rather than universal properties of ZnSe(Al,O). ZnSe-based scintillators are strongly affected by dopant concentration, defect structure, and crystal growth quality. Direct characterisation is therefore important when reliable parameters are required for quantitative detector modelling. Although this study investigated a single crystal, the experimental framework is not specific to ZnSe(Al,O). The same approach can be applied to other scintillator materials where manufacturer data are incomplete and can provide experimentally constrained inputs for detector design and simulation.

For the present detector application, the measured light yield of approximately $7.69\times 10^{4}$ photons ${\mathrm{MeV}}^{-1}$, decay time of 9.41 μs, and emission peak near 581 nm support the use of ZnSe(Al,O) with SiPM readout for beta spectroscopy. The resulting parameter set is directly relevant to the development of the intended detector for in situ Sr-90 monitoring in groundwater. Future work will use these experimentally verified values to refine Geant4 optical modelling and optimise the optical coupling and electronic signal conditioning of further detector prototypes.

## Figures and Tables

**Table 1:** Summary of reported detector properties for ZnSe-based scintillators, including refractive index, light yield, decay time, density, emission wavelength and dopant concentrations.

Scintillator	Refractive Index	Light Yield (Photons/MeV)	Decay (μs)	Density (${\mathrm{g cm}}^{-3}$)	Peak Emission (nm)	Dopant Concentrations (Mass%)
ZnSe(Te)	^1^ 2.61, ^2^ 2.59 [7] 2.61 [9]	$7\times 10^{4}$ [4,10] ^1^ $6\times 10^{4}$, ^2^ $7.5\times 10^{4}$ [7] $7\times 10^{4}$ [11] $4\times 10^{4}$	30–80 [4,10] ^1^ 1–3, ^2^ 30–70 [7] 0.02 [12]	5.42 [12]	640 [4,10] ^1^ 610, ^2^ 640 [7] ^3^ 640, ^4^ 630 & 465 [8] 640 [12]	0.2 [4] 1 [6] 0.3–0.6 [9]
ZnSe(O)	–	$5\times 10^{4}$ [4,10]	2–8 [4,10] ^5^ 9, 28; ^6^ 4, 12 [5]	–	600 [4,10] 595–600 [5]	0.02 [4] 2 [5]
ZnSe(Te,O)	–	–	5–7 3–5 [13]	–	620–640 [13]	–
ZnSe(Al)	2.6 [3] 2.58 [9]	$8\times 10^{4}$ [9]	1–3 [9]	5.42 [3]	^3^ 600, ^4^ 630 & 550 [8] 610 [3]	–
ZnSe(Al,O)	–	$5\times 10^{4}$ [4,10]	3–15 [4,10]	–	605 [4,10] ^7^ 575–620, ^8^ 640–650 [6]	^9^ 0.03–0.1 [6]
ZnSe(Cd)	–	$4\times 10^{4}$ [4,10]	5–20 [4,10]	–	620 [4,10]	–
ZnSe(Cd,Te)	–	$6\times 10^{4}$ [4,10]	40–100 [4,10]	–	635 [4,10]	–

^1^ Fast component. ^2^ Slow component. ^3^ Peak emission at 295 K. ^4^ Peak emission at 77 K. ^5^ As-grown crystal. ^6^ Annealed crystal. ^7^ High-energy emission band. ^8^ Low-energy emission band. ^9^ Doped with ${\mathrm{Al}}_{2}O_{3}$.

**Table 2:** A table showing the relative atomic abundance of elements present in the ZnSe(Al,O) scintillator from EDX analysis.

Element	Atomic Abundance I (%)	Atomic Abundance II (%)	Average Atomic Abundance (%)
Zn	51.4	49.2	50.3
Se	41.9	46.1	44.0
O	5.5	3.5	4.5
Al	1.2	1.2	1.2

## Data Availability

Data of this research is available upon request via first author.
